# Common Variants of *FTO* Are Associated with Childhood Obesity in a Cross-Sectional Study of 3,126 Urban Indian Children

**DOI:** 10.1371/journal.pone.0047772

**Published:** 2012-10-16

**Authors:** Om Prakash Dwivedi, Rubina Tabassum, Ganesh Chauhan, Saurabh Ghosh, Raman K. Marwaha, Nikhil Tandon, Dwaipayan Bharadwaj

**Affiliations:** 1 Genomics and Molecular Medicine Unit, CSIR-Institute of Genomics and Integrative Biology, Delhi, India; 2 Human Genetics Unit, Indian Statistical Institute, Kolkata, India; 3 Department of Endocrinology and Thyroid Research, Institute of Nuclear Medicine and Allied Sciences, Delhi, India; 4 Department of Endocrinology, All India Institute of Medical Sciences, New Delhi, India; Sanjay Gandhi Medical Institute, India

## Abstract

**Background:**

*FTO* variants are robustly associated with obesity and related traits in many population and shown to have variable impact during life course. Although studies have shown association of *FTO* variants with adiposity in adult Indian, its association in Indian children is yet to be confirmed.

**Methods:**

Here we examined association of *FTO* variants (rs9939609 and rs8050136) with obesity and related anthropometric and biochemical traits in 3,126 Indian children (aged 11–17 years) including 2,230 normal-weight and 896 over-weight/obese children. We also compared effects observed in the present study with that observed in previous studies on South Asian adults and children of other ethnic groups.

**Results:**

The variant rs9939609 showed significant association with risk of obesity [OR = 1.21, *P* = 2.5×10^−3^] and its measures BMI, weight, waist circumference and hip circumference [β range = 0.11 to 0.14 Z-score units; *P* range = 1.3×10^−4^ to 1.6×10^−7^] in children. The observed effect sizes in Indian children were similar to those reported for European children. Variant rs9939609 explained 0.88% of BMI variance in Indian children. The effect sizes of rs9939609 on BMI and WC were ∼2 fold higher in children than adults. Interestingly rs9939609 was also associated with serum levels of thyroid stimulating hormone (TSH) [β = 0.10 Z-score, *P* = 5.8×10^−3^]. The other variant rs8050136 was in strong linkage disequilibrium with rs9939609 (r^2^ = 0.97) and provided similar association results.

**Conclusion:**

The study provides first report of association of *FTO* variants with obesity and related anthropometric traits in Indian children with higher impact in children compared to adults. We also demonstrated association of *FTO* variant with serum levels of TSH, indicating putative influence of *FTO* in hypothalamic-pituitary-thyroid axis.

## Introduction

Genome wide association studies (GWAS) have revolutionized the discovery of obesity-susceptibility loci at population level in last five years. Till date, at least 52 loci associated with obesity risk and obesity-related traits have been identified through GWAS [1]. Fat mass and obesity associated (*FTO*) gene was the first obesity associated locus implicated by GWAS [2], [3]. Among all the GWAS-identified obesity associated loci, *FTO* variants have strongest influence on obesity and contribute maximally to the variance in body mass index (BMI) in Europeans (0.34%) and East Asians (0.18%) [4], [5]. Since its discovery, association of *FTO* locus has been demonstrated in adults and children from different ethnicities, not only with BMI but also with the risk of obesity, body fat percentage, waist circumference (WC) and other related traits [6].

The human genetic association studies of *FTO* with obesity are further supported by subsequent functional studies in animal models. In mice, loss of function and/or expression of *Fto* leads to lean phenotype while its overexpression results in obesity [7], [8]. Studies have shown that *FTO* functions as a demethylase and predominantly affect obesity by influencing energy intake [7]–[9].

*FTO* variants are suggested to have variable impact during the life course [10]–[12], though these reports have been inconsistent across population. A study in Europeans suggested stronger impact of *FTO* variants on BMI during childhood and adolescence compared to adulthood, while another study showed positive association of risk allele of *FTO* variant with BMI in childhood but a negative association during infancy [10], [11]. As *FTO* variants have varied longitudinal effect, association studies across the age groups are essential to comprehensively evaluate its influence in modulating the risk of obesity in a population. Although association of *FTO* variants with adult obesity in Asian population has been confirmed by many studies, investigation of its effect on childhood obesity is limited particularly in South Asians [13]–[16]. Among South Asians only one study has examined association of *FTO* variant with obesity during childhood from Southern part of India and failed to detect any association at younger ages [12].

Here we examined two variants rs9939609 and rs8050136 from first intron of *FTO*, which are most robustly implicated in obesity, for associations with obesity and related traits in 3,126 Indian children. We also compared their effects on obesity risk in Indian children with that in South Asian adults and children from other ethnic groups. The present study would enable us to understand influence of *FTO* variants during early life in Indian population that are at higher risk to develop obesity in adult life.

**Table 1:** Anthropometric and clinical characteristic of study subjects.

Character	All children	NW children	OW/OB children
N (Boys/Girls)	3126 (1094/2032)	2230 (789/1441)	896 (305/591)
Age (years)*	13.50 (1.88)	13.51 (1.85)	13.45 (1.95)
Height (cm)*	154.21 (9.90)	153.28 (9.93)	156.52 (9.43)
Weight (Kg)*	49.00 (14.29)	42.61 (8.96)	64.92 (12.52)
BMI (Kg/m^2^)*	20.37 (4.68)	17.97 (2.46)	26.32 (3.41)
WC (cm)*	71.10 (11.64)	66.10 (7.76)	84.27 (9.71)
HC (cm)*	85.24 (11.11)	80.57 (7.74)	97.43 (9.14)
WHR*	0.83 (0.07)	0.82 (0.06)	0.87 (0.07)
Total cholesterol (mg/dl)^ †^	142.00 (127.00–162.55)	138.20 (124.00–156.15)	154.00 (134.25–176.00)
HDL-C (mg/dl)^ †^	45.00 (40.00–49.89)	45.00 (41.00–51.00)	43.00 (38.40–48.00)
LDL-C (mg/dl)^ †^	84.00 (71.00–95.40)	82.00 (69.10–92.20)	90.00 (75.93–106.90)
TG (mg/dl)^ †^	95.00 (72.00–127.00)	93.00 (72.00–123.00)	103.00 (71.00–142.00)
Fasting Glucose (mg/dl)^ †^	87.20 (81.00–94.00)	88.00 (81.85–94.00)	86.80 (80.05–93.00)
Fasting Insulin (pmol/L)^ †^	46.80 (30.30–71.38)	40.08 (26.34–57.78)	74.40 (48.30–110.25)
HOMA-IR^ †^	1.66 (1.07–2.52)	1.44 (0.94–2.09)	2.60 (1.66–3.97)
TSH (mIU/l)^ †^	2.90 (2.13–4.09)	2.90 (2.12–4.06)	2.90 (2.18–4.15)
FT4 (pmol/L)^ †^	15.57 (14.14–17.11)	15.78 (14.31–17.34)	15.02 (13.55–16.35)
FT3 (pmol/L)^ †^	4.77 (4.24–5.32)	4.78 (4.25–5.36)	4.70 (4.22–5.28)
Leptin (ng/mL)^ †^	9.62 (5.44–16.03)	7.66 (4.72–12.24)	18.38 (11.69–28.57)
Adiponectin (ng/mL)^ †^	7.80 (4.63–12.09)	8.53 (5.27–13.09)	5.67 (3.37–9.22)
Resistin (ng/mL)^ †^	5.50 (4.34–7.15)	5.40 (4.30–7.10)	5.73 (4.48–7.25)

* Data presented as mean ± standard deviation; †Data presented as median (interquartile range); N: Number of subjects; NW: normal weight children; OW/OB: overweight and obese children; HC: hip circumference; WC: waist circumference; WHR: waist-hip ratio; HDL-C: high density lipoprotein-cholesterol; LDL-C: low density lipoprotein-cholesterol; HOMA-IR: homeostasis model assessment of insulin resistance; TSH: thyroid stimulating hormone; FT4: free thyroxine; FT3: free tri-iodothyronine. Data for serum levels of TSH, FT3 and FT4 was available only for 1,822 subjects.

**Table 2:** Association of *FTO* variants with obesity and metabolic traits in Indian children.

	rs9939609					rs8050136				
Traits	TT	TA	AA	OR (95%CI)	*P*	CC	CA	AA	OR (95%CI)	*P*
Obesity	N (frequency)					N (frequency)				
NW children	985 (0.45)	935 (0.44)	227 (0.11)	1.00		980 (0.45)	944 (0.44)	236 (0.11)	1.00	
OW/OB children	347 (0.41)	377 (0.44)	124 (0.15)	1.21 (1.07–1.37)	**2.5×10^−3^**	351 (0.40)	397 (0.45)	126 (0.15)	1.19 (1.05–1.35)	**5.0×10^−3^**

Quantitative traits		Mean (SD)		β (95% CI)	*P*		Mean (SD)		β (95% CI)	*P*
Z-Height	0.00 (0.99)	0.00 (1.00)	0.01 (1.00)	0.00 (−0.05–0.05)	0.88	0.00 (0.99)	0.02 (0.99)	0.01 (0.99)	0.00 (−0.05–0.05)	0.97
Z-Weight	−0.05 (1.01)	0.04 (0.99)	0.17 (0.98)	0.12 (0.06–0.17)	**1.1×10^−5^**	−0.05 (1.01)	0.06 (0.99)	0.16 (0.98)	0.12 (0.06–0.17)	**1.4×10^−5^**
Z-BMI	−0.07 (1.02)	0.05 (0.99)	0.21 (0.96)	0.14 (0.09–0.19)	**1.6×10^−7^**	−0.06 (1.02)	0.07 (0.99)	0.19 (0.96)	0.14 (0.08–0.19)	**4.2×10^−7^**
Z-WC	−0.07 (1.02)	0.03 (0.99)	0.16 (0.95)	0.12 (0.07–0.17)	**1.7×10^−5^**	−0.07 (1.03)	0.06 (0.99)	0.16 (0.94)	0.12 (0.07–0.18)	**7.9×10^−6^**
Z-HC	−0.04 (0.99)	0.04 (1.01)	0.16 (0.97)	0.11 (0.05–0.16)	**1.3×10^−4^**	−0.04 (0.99)	0.06 (1.02)	0.15 (0.97)	0.11 (0.05–0.16)	**1.5×10^−4^**
Z-WHR	−0.05 (1.03)	0.01 (0.96)	0.08 (0.95)	0.06 (0.01–0.12)	**0.02**	−0.06 (1.04)	0.03 (0.97)	0.07 (0.94)	0.07 (0.02–0.13)	**9.2×10^−3^**
Z-Total Cholesterol	−0.05 (0.99)	−0.01 (0.98)	0.02 (1.05)	0.01 (−0.04–0.06)	0.74	−0.05 (0.99)	0.00 (0.99)	0.01 (1.04)	0.01 (−0.04–0.06)	0.72
Z-HDL-C	0.02 (0.98)	−0.03 (1.00)	−0.02 (1.00)	−0.01 (−0.06–0.05)	0.82	0.01 (0.98)	−0.02 (1.00)	−0.03 (1.01)	0.00 (−0.05–0.05)	0.93
Z-LDL-C	−0.03 (0.99)	0.01 (0.97)	0.02 (1.04)	0.0 (−0.05–0.05)	0.93	−0.03 (0.98)	0.02 (0.98)	0.03 (1.03)	0.00 (−0.05–0.06)	0.85
Z-TG	0.01 (0.97)	0.08 (0.95)	0.11 (1.04)	0.03 (−0.02–0.09)	0.19	0.02 (0.97)	0.06 (0.96)	0.12 (1.05)	0.03 (−0.02–0.08)	0.25
Z-FPG	−0.02 (1.01)	0.09 (0.98)	−0.05 (1.03)	0.03 (−0.02–0.09)	0.25	−0.03 (1.01)	0.06 (0.98)	−0.02 (1.03)	0.04 (−0.02–0.09)	0.16
Z-FPI	−0.02 (0.99)	−0.01 (1.02)	0.10 (0.96)	−0.02 (−0.07–0.03)	0.39	−0.02 (0.99)	0.01 (1.03)	0.10 (0.96)	−0.01 (−0.06–0.04)	0.65
Z-HOMA-IR	−0.02 (0.99)	0.00 (1.02)	0.09 (0.97)	−0.01 (−0.06–0.04)	0.61	−0.03 (0.99)	0.02 (1.03)	0.09 (0.97)	0.00 (−0.05–0.05)	0.95
Z-Leptin	−0.03 (0.98)	0.01 (1.00)	0.17 (0.98)	0.0 (−0.04,0.05)	0.92	−0.02 (0.98)	0.04 (1.00)	0.14 (0.98)	0.0 (−0.04,0.05)	0.89
Z-Adiponectin	0.02 (0.99)	0.02 (0.98)	−0.03 (1.02)	0.02 (−0.04,0.07)	0.54	0.01 (0.98)	0.02 (0.98)	−0.03 (1.03)	0.02 (−0.03,0.07)	0.41
Z-Resistin	0.01 (0.99)	0.0 (0.99)	0.04 (1.06)	0.0 (−0.06,0.05)	0.92	0.0 (0.99)	0.0 (1)	0.03 (1.06)	0.0 (−0.05,0.06)	0.87
Z-FT3^‡^	−0.07 (0.99)	−0.04 (0.95)	0.00 (1.01)	0.04 (−0.03–0.11)	0.30	−0.07 (0.99)	−0.04 (0.94)	0.01 (1.00)	0.04 (−0.03–0.11)	0.26
Z-FT4^‡^	0.00 (1.03)	−0.05 (0.94)	−0.07 (1.08)	−0.01 (−0.08–0.06)	0.73	0.00 (1.02)	−0.06 (0.94)	−0.04 (1.08)	0.00 (−0.07–0.06)	0.92
Z-TSH^‡^	−0.07 (0.99)	0.07 (0.97)	0.08 (1.01)	0.10 (0.03–0.17)	**5.8×10^−3^**	−0.07 (0.99)	0.08 (0.97)	0.06 (1.01)	0.09 (0.02–0.16)	**8.4×10^−3^**

N: Number of subjects; SD: standard deviation; CI: confidence interval; OR: odds ratio with respect to minor allele; HC: hip circumference; WC: waist circumference; WHR: waist-hip ratio; HDL-C: high density lipoprotein-cholesterol; LDL-C: low density lipoprotein-cholesterol; HOMA-IR: homeostasis model assessment of insulin resistance; TSH: thyroid stimulating hormone; FT4: free thyroxine; FT3: free Tri-iodothyronine. β represents change in Z score unit per increase in minor allele. ‡ Association analysis was performed in 1,765 successful genotyped subjects.

## Materials and Methods

### Ethics Statement

Prior informed written consent was obtained from parents/guardians of the children while verbal consent from children themselves was taken. The study was approved by the Human Ethics Committee of CSIR-Institute of Genomics and Integrative Biology, the All India Institute of Medical Sciences Research Ethics Committee and the Ethics Committee of the Institute of Nuclear Medicine and Allied Sciences. The study was conducted in accordance with the principles of Helsinki Declaration.

**Figure 1:**
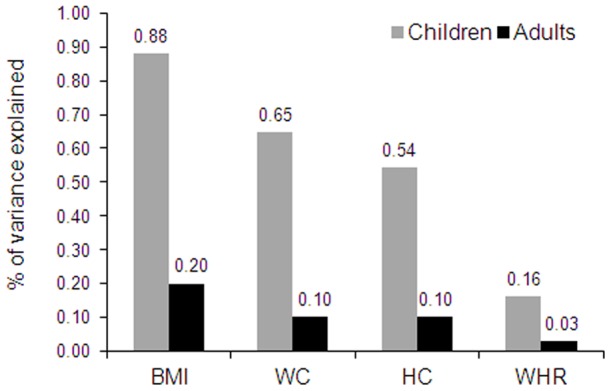
Variance in adiposity measures explained by *FTO* variant rs9939609 in Indian children and adults. BMI: body mass index; WC: waist circumference; HC: hip circumference; WHR: waist-to-hip ratio. Data for children were taken from present study while the data for adults were taken from a recent meta-analysis (up to 17,124 subjects) on South Asian adults by *Li et* *al* [13].

### Subjects

The study included a total of 3,126 school children from both the sexes in the age group of 11–17 years. The children were recruited as a part of ongoing health survey of government and private schools located in four different geographical zones of Delhi (India), as described previously [17]. Using age and sex specific BMI cut-offs provided by International Obesity Task Force (IOTF), the recruited children were categorized as normal-weight (N = 2230) and over-weight/obese (N = 896) [18].

**Table 3:** Comparison of effect sizes of rs9939609 for adiposity measures.

	Indian Children* (N = 3,126)	Indian Adults† (N = up to 17,124)	Q	I^2^	Indian Children* (N = 3,126)	European Children ‡ (N = up to19,268)Children‡(Up to 19,268 children)	Q	I^2^
Traits	β (95%CI)	β (95%CI)			β (95%CI) (In Z-score units)	β (95%CI) (In Z-score units)		
BMI (kg/m^2^)	0.64 (0.41–0.88)	0.29 (0.13–0.44)	0.02	82.42	0.14 (0.09–0.19)	0.10 (0.08–0.12)	0.16	48.60
WC (cm)	1.27 (0.65–1.89)	0.54 (0.28–0.81)	0.03	77.86	0.12 (0.07–0.17)	0.11 (0.08–0.13)	0.75	0.00
HC (cm)	1.06 (0.50–1.63)	0.46 (0.13–0.79)	0.07	68.88	-	-	-	-
Z-WHR	0.004 (0.000–0.007)	0.002 (0.001–0.004)	0.28	15.49	-	-	-	-

β represents change in Z score unit or in the respective units of traits with per increase in minor allele. N: Number of subjects; Q: *P* value for Cochrane's Q statistic for heterogeneity of effects; I^2^: Î2 heterogeneity index (0–100). *Summary statistic data for obesity measures were taken from present study, †Summary statistic data for obesity measures were taken from recent meta-analysis on south Asian adults by *Li et* *al*. [13], ‡Summary statistic data for obesity measures were taken from recent meta-analysis on European children by *Kilpeläinen et* *al*. [24].

### Measurements

All the subjects were extensively characterized for various anthropometric and clinical traits as described previously [19], [20]. Height, weight, WC and hip circumference (HC) were measured using standard methods. BMI and waist-to-hip ratio (WHR) were calculated using anthropometric measures. Fasting plasma levels of glucose, high sensitivity C-reactive protein (hsCRP), total cholesterol (TC), high density lipoprotein-cholesterol (HDL-C), low density lipoprotein cholesterol (LDL-C), triglyceride (TG) were measured using Cobas Integra 400 Plus (Roche Diagnostics, GmbH, Mannheim, Germany). Plasma levels of insulin were estimated using Elecsys 2010 (Roche Diagnostics). Plasma levels of leptin, resistin and adiponectin were estimated using commercial ELISA kits (R&D Systems, Minneapolis, MN, USA). Serum levels of free tri-iodothyronine (FT3), free thyroxin (FT4) were estimated by radio immunoassay and thyroid stimulating hormone (TSH) by immunoradiometric assay (Immunotech, Beckman Coulter).

### Genotyping

Genotyping of the variants was performed using Illumina GoldenGate assay (Illumina Inc., San Diego, CA, USA) and iPLEX assay (Sequenom San Diego, CA, USA). Stringent quality control (QC) was applied to the genotyped data (Table S1) as discussed previously [21]. The genotype call rate was >95% for both the variants. Genotype distributions of both variants followed Hardy Weinberg Equilibrium (HWE).

### Statistical analyses

Statistical analyses were performed using PLINK v.1.07 (http://pngu.mgh.harvard.edu/purcell/plink) [22] and SPSS v. 17.0 (SPSS, Chicago, IL, USA). Genotype distributions were tested for HWE through χ^2^analysis. Pairwise linkage disequilibrium (LD) between SNPs was determined using Haploview 4.0 software [23]. Association of variants with obesity was tested using logistic regression under additive model adjusting for age and sex. Additionally, we also tested association of variants with obesity risk under dominant and recessive models adjusting for age and sex. Continuous quantitative traits were transformed to normal distribution using inverse normal transformation. Prior to analysis, continuous variables were converted to age and sex specific internal Z-scores by dividing differences of individual values and mean values of study population by standard deviation. Association of variants with height, weight, WC, HC, BMI and WHR were assessed by linear regression using additive model adjusting for age and sex while associations with other continuous variables were adjusted for age, sex and Z-BMI. Effect sizes were expressed as change in Z-score unit. We also estimated per allele effect size in their respective units that are compatible with other reported studies used for comparison. Heterogeneity in effect sizes was estimated using Cochran's Q statistics. Percentage of variance explained by *FTO* variants was quantified using the equation 2f (1-f)β^2^, where f is frequency of variant in population and β is standardized additive effect size [4].

## Results

Anthropometric and clinical characteristics of the subjects are provided in Table 1. Both variants rs9939609 and rs8050136 were in strong LD with each other (r^2^ = 0.97). As two SNPs represent same signal and association results were similar (Table 2), all results have been discussed with respect to rs9939609.

The rs9939609 showed significant association with risk of obesity in children [OR = 1.21, *P* = 2.5×10^−3^ for additive model] (Table 2). Consistently, we also observed significant association of the risk allele (Table 2) with increase in BMI [β = 0.14 Z-score unit, *P* = 1.6×10^−7^], weight [β = 0.12, *P* = 1.1×10^−5^], WC [β = 0.12, *P* = 1.7×10^−5^] and HC [β = 0.11, *P* = 1.3×10^−4^]. We also performed association analysis assuming dominant and recessive genetic models. Association result for variant rs9939609 under dominant model [OR = 1.23, *P* = 1.2×10^−2^] was similar to additive model [OR = 1.21, *P* = 2.5×10^−3^]. However, we observed slightly higher effect size under recessive model [OR = 1.46, *P* = 1.8×10^−3^] compared to additive and dominant models. Variant rs9939609 explains 0.65% to 0.88% of the inter-individual variation in measures of obesity (Figure 1). Further, we explored gender differences in the effect of *FTO* variants on obesity measures. We did not find any significant difference in the effect of *FTO* variants among boys and girls for any anthropometrical traits (Table S2).

Further, to investigate age dependent influence of *FTO* variants on obesity traits, we compared the effect sizes of rs9939609 on adiposity parameters in children with that of adults from a recent and largest meta-analysis on South Asian adults (up to 17,124 adults) [13]. Significant heterogeneity in the effects of rs9939609 (I^2^>77.86%) between children and adults on BMI [0.64 kg/m^2^ for children and 0.29 kg/m^2^ for adults] and WC [1.27 cm for children and 0.54 cm for adults] were found (Table 3). The effect size on WHR between children and adults were similar with low heterogeneity (I^2^ = 15.49%). Further we compared the effect of variant rs9939609 on obesity traits in children with that of adults from North India from our previous study [16]. Consistently, we observed high heterogeneity in effect size on BMI in children compared to the adults (I^2^ = 67.2%). However, there was no difference (I^2^ = 0) in effect sizes of WC and WHR between North Indian children and adults (Table S3).

*FTO* variants are shown to have varying effect on adiposity measures among adults in different ethnic groups [6]. Comparison of influences of *FTO* variants on BMI and WC in Indian children from present study and European children from a meta-analysis study (up to 19,268 children) [24] showed only low to moderate heterogeneity (I^2^ = 0 to 48.6%) (Table 3). Similarly the observed effect of *FTO* variant on BMI in Indian children (0.64 kg/m^2^) was similar to those reported for East Asian children (0.29 to 0.50 kg/m^2^) [14], [15].

Next, we performed exploratory analysis to investigate association of *FTO* variants with biochemical markers of glucose metabolism, lipid metabolism, thyroid function and inflammation as all the parameters are related to obesity. Intriguingly, we observed significant association of minor allele of rs9939609 with elevated serum levels of TSH in children [β = 0.10, *P* = 5.8×10^−3^] (Table 2). We also found BMI dependent association of rs9939609 with plasma level of leptin [β = 0.08, *P* = 4.0×10^−3^], however that diminished after adjusting for BMI (Table 2). We did not observe any association of rs9939609 with makers of glucose and lipid metabolism (Table 2).

## Discussion

Numerous GWAS and subsequent replication studies in distinct population of European, African and Asian origin have robustly established association of *FTO* with obesity parameters, both in adults and children [6]. The present study evaluated the effect of *FTO* variants (rs9939609 and rs8050136) on susceptibility to obesity and related traits in Indian children. To the best of our knowledge, this is the first report demonstrating association of *FTO* variant with obesity in children from South Asia.

### Influence of *FTO* variants on adiposity measures

*FTO* variants showed strong influence on overall adiposity in Indian children. Children homozygous for minor allele of rs9939609 had ∼1.5 kg/m^2^ higher BMI, ∼2 cm higher WC and ∼2 kg higher weight compared to children homozygous for other allele. The effect sizes of the variants on BMI, weight, WC and HC were similar (β range 0.11 to 0.14 Z-score), indicating similar influence on the measures of adiposity. Interestingly, a recent study (Vasan et al.) on longitudinal birth cohort from South India [12] showed association of rs9939609 with obesity-related traits in adulthood, but not at younger ages. It is noteworthy that present study differ in sample size, study design and subject recruitment from the study by Vasan et al that has lower samples size (up to 1,644 in adolescence) and recruited subjects from rural and urban regions of South India. Environmental factors including diet and physical activity are known to modulate the effect of *FTO* variants on adiposity [24], [25]. The study (Vasan et al.) itself showed effect of *FTO* on obesity traits is influenced by urban living conditions. The observed discrepancy in the association results might be due to lower statistical power contributed by smaller sample size and attenuated influence of *FTO* on adiposity in recruited subjects.

### Age dependent influence of *FTO* variants on adiposity measures

*FTO* variants observed effect sizes and contribution in variance of adiposity traits in Indian children are higher than South Asian adults. Per minor allele increase in BMI is ∼2 fold higher in children compared to adults. Further, the genetic contribution of *FTO* variants on BMI variance in children (0.88%) is almost four times higher to those reported for adult BMI variance (0.20%) in South Asian [13]. Similarly, for other adiposity parameters (WC and WHR) too, *FTO* variants have higher contribution to the variance in children (0.54–0.65%) than South Asian adults (0.03–0.10%). Thus our results clearly indicate that *FTO* variants have biphasic effect with greater impact during childhood when compared to adulthood. The age related changes in lifestyle, eating behavior and exposure to environmental factors could be possible reasons for the age dependent variation in effect of *FTO* variants on obesity related anthropometric traits. However, the present study design does not allow investigating such gene-environment interaction. Further studies with information on eating behavior and environmental factors could provide better insight to it.

### Comparing effect sizes in Indian children with children from other population

The comparison of effect sizes of *FTO* variants on adiposity measures between Indian and European children revealed its similar influence on both population despite the evident variation in their anthropometric features and genetic architecture. The effect of *FTO* variants on obesity parameters are shown to be generally smaller in Asian adults compared to European adults [13]. However, we found similar effect of *FTO* variants on adiposity measures in children from Indian, European and East Asian population. This suggests that *FTO* variants has uniform and equal influence during childhood in different ethnic population but varies later in life.

### Influence of *FTO* variants on serum TSH levels

The understanding of molecular mechanism of *FTO* in weight regulation is just started to begin and largely remains elusive till date. *FTO* predisposes to weight gain primarily due to increase in energy intake, however animal studies have also shown its effect on metabolic rate and energy expenditure [7], [8]. Further *FTO* has also been shown to have BMI independent effect on metabolic traits [26]. Our study demonstrates association of *FTO* variants with levels of TSH that is produced in pituitary gland. *FTO* is also abundantly expressed in pituitary gland. Previous studies have shown that slight change in TSH levels is associated with weight gain and it directly correlates with fat mass [27]. The association of *FTO* risk allele with increased levels of TSH as well as its BMI dependent association with leptin levels indicates putative involvement of *FTO* in hypothalamic-pituitary-thyroid axis in mediating metabolic effects.

### Limitations of present study

Though the present study indicates influence of *FTO* variants on pituitary function through association with TSH levels, the possibility of reverse causation (i.e. increase in TSH level as a result of obesity caused by *FTO* variants) could not be completely ruled out due to cross-sectional design of the study. Further studies are warranted in this direction to confirm and elucidate the precise role of *FTO* in pituitary function.

### Conclusion

We demonstrate here, for the first time, association of *FTO* variants with obesity risk and adiposity measures (BMI, weight, WC and HC) in Indian children. The study also suggests that *FTO* variants have age dependent influence on adiposity traits in Indians with higher impact in children compared to adults.

## Supporting Information

Table S1 **Quality check for genotyped data.**
(DOC)

Table S2 **Comparison of effect sizes of** ***FTO*** **variant rs9939609 on adiposity measures between Indian boys and girls.** β represents change in Z score per increase in minor allele; CI: confidence interval; Q: *P* value for Cochrane's Q statistic; I: Î2 heterogeneity index (0–100).
(DOC)

Table S3 **Comparison of effect sizes of** ***FTO*** **variant on adiposity measures in North Indian children and adults.** β represents per minor allele change in trait. Q: *P* value for Cochrane's Q statistic for heterogeneity of effects; I^2^: Î2 heterogeneity index (0–100). *Summary statistic data for obesity measures were taken from present study, † Summary statistic data for obesity measures were taken from study on North Indian adults (up to 2,626 adults) by *Chauhan et* *al* [16].
(DOC)
