# Supplementary material for: Common Variants of FTO Are Associated with Childhood Obesity in a Cross-Sectional Study of 3,126 Urban Indian Children
Source: PLoS One. 2012 Oct 16;7(10):e47772. doi: 10.1371/journal.pone.0047772 (PMC3472993; doi:10.1371/journal.pone.0047772)
Supplement: Table S1 — Quality check for genotyped data. (DOC) [file pone.0047772.s001.doc]

**Table S1: Quality check for genotyped data.**

| **SNP** | **Base change** | **Call rate**  **(%)** | ***P* value for Hardy Weinberg Equilibrium** | | |
| --- | --- | --- | --- | --- | --- |
|  |  |  | **Normal-weight children** | **Overweight/obese children** | **All children** |
| rs9939609 | T/A | 95.81 | 0.81 | 0.21 | 0.31 |
| rs8050136 | C/A | 97.06 | 0.70 | 0.43 | 0.40 |
