# Supplementary material for: Common Variants of FTO Are Associated with Childhood Obesity in a Cross-Sectional Study of 3,126 Urban Indian Children
Source: PLoS One. 2012 Oct 16;7(10):e47772. doi: 10.1371/journal.pone.0047772 (PMC3472993; doi:10.1371/journal.pone.0047772)
Supplement: Table S2 — Comparison of effect sizes of FTO variant rs9939609 on adiposity measures between Indian boys and girls. β represents change in Z score per increase in minor allele; CI: confidence interval; Q: P value for Cochrane's Q statistic; I: Î2 heterogeneity index (0–100). (DOC) [file pone.0047772.s002.doc]

**Table S2. Comparison of effect sizes of *FTO* variant rs9939609 on adiposity measures between Indian boys and girls.**

|  | **β (95% CI) in Z-score units** | |  |  |
| --- | --- | --- | --- | --- |
| **Trait** | **Boys** | **Girls** | **I^2^** | **Q** |
| Z-Weight | 0.08 (-0.01,0.17) | 0.12 (0.06-0.19) | 0.0 | 0.89 |
| Z-BMI | 0.12 (0.03-0.21) | 0.14 (0.08-0.21) | 0.0 | 0.86 |
| Z-HC | 0.09 (0.00-0.18) | 0.10 (0.03-0.17) | 0.0 | 0.75 |
| Z-WC | 0.10 (0.01-0.19) | 0.12 (0.05-0.19) | 0.0 | 0.44 |
| Z-WHR | 0.06 (-0.03,0.15) | 0.07 (0.00-0.14) | 0.0 | 0.71 |

β represents change in Z score per increase in minor allele; CI: confidence interval; Q: *P* value for Cochrane's Q statistic; I: I^2 heterogeneity index (0-100)
