# Supplementary material for: Common Variants of FTO Are Associated with Childhood Obesity in a Cross-Sectional Study of 3,126 Urban Indian Children
Source: PLoS One. 2012 Oct 16;7(10):e47772. doi: 10.1371/journal.pone.0047772 (PMC3472993; doi:10.1371/journal.pone.0047772)
Supplement: Table S3 — Comparison of effect sizes of FTO variant on adiposity measures in North Indian children and adults. β represents per minor allele change in trait. Q: P value for Cochrane's Q statistic for heterogeneity of effects; I2: Î2 heterogeneity index (0–100). *Summary statistic data for obesity measures were taken from present study, † Summary statistic data for obesity measures were taken from study on North Indian adults (up to 2,626 adults) by Chauhan et al [16]. (DOC) [file pone.0047772.s003.doc]

**Table S3: Comparison of effect sizes of *FTO* variant on adiposity measures in Indian children and Indian adults.**

|  | **β (95%CI)** | |  |  |
| --- | --- | --- | --- | --- |
| **Traits** | **Indian Children***  **(N=3,126)** | **North Indian adults†**  **(Up to 2626 adults)** | **Q** | **I^2^** |
| BMI (kg/m^2^) | 0.64 (0.41-0.88) | 0.32 (0.06-0.59) | 0.08 | 67.2 |
| WC (cm) | 1.27 (0.65-1.89) | 1.02 (0.38-1.66) | 0.58 | 0.0 |
| Z-WHR | 0.004 (0.000-0.007) | 0.004 (0.000-0.008) | 1.00 | 0.0 |

β represents per minor allele change in trait. Q: *P* value for Cochrane's Q statistic for heterogeneity of effects; I^2^: I^2 heterogeneity index (0-100). *Summary statistic data for obesity measures were taken from present study, † Summary statistic data for obesity measures were taken from study on north Indian adults (up to 2,626 adults) by *Chauhan et al* [14].
